# Rapid Visual Detection of Hepatitis C Virus Using Reverse Transcription Recombinase-Aided Amplification–Lateral Flow Dipstick

**DOI:** 10.3389/fcimb.2022.816238

**Published:** 2022-02-17

**Authors:** Haili Wang, Yuhang Zhang, Jingming Zhou, Ming Li, Yumei Chen, Yankai Liu, Hongliang Liu, Peiyang Ding, Chao Liang, Xifang Zhu, Ying Zhang, Cheng Xin, Gaiping Zhang, Aiping Wang

**Affiliations:** ^1^ School of Life Sciences, Zhengzhou University, Zhengzhou, China; ^2^ College of Veterinary Medicine, Henan Agricultural University, Zhengzhou, China; ^3^ Henan Key Laboratory of Population Defects Prevention, National Health Commission Key Laboratory of Birth Defects Prevention, Henan Institute of Reproduction Health Science and Technology, Zhengzhou, China

**Keywords:** hepatitis C virus, nucleic acid detection, point-of-care testing, recombinase-aided amplification, lateral flow dipstick

## Abstract

Hepatitis C virus (HCV) infection is a global public health threat. Reaching the World Health Organization’s objective for eliminating viral hepatitis by 2030 will require a precise disease diagnosis. While immunoassays and qPCR play a significant role in detecting HCV, rapid and accurate point-of-care testing is important for pathogen identification. This study establishes a reverse transcription recombinase-aided amplification–lateral flow dipstick (RT-RAA-LFD) assay to detect HCV. The intact workflow was completed within 30 min, and the detection limit for synthesized C/E1 plasmid gene-containing plasmid was 10 copies/μl. In addition, the test showed good specificity, with no cross-reactivity observed for hepatitis A virus, hepatitis B virus, HIV, syphilis, and human papillomavirus virus. Using extracted RNAs from 46 anti-HCV antibody-positive samples, RT-RAA-LFD showed 100% positive and negative concordance rates with qPCR. In summary, the RT-RAA-LFD assay established in this study is suitable for the rapid clinical detection of HCV at the community level and in remote areas.

## Introduction

Infectious diseases pose a critical global threat that has caused significant morbidity and mortality as well as great economic losses (Houghton, 2009; Massimo and Vincenzo, 2018). Hepatitis C virus (HCV) infection is a major cause of severe liver diseases, including chronic hepatitis, cirrhosis, and hepatocellular carcinoma. According to the World Health Organization (WHO, 2017), approximately 71 million people worldwide are infected with HCV, and at least 400,000 people die annually from HCV-related liver disease (WHO, 2017; Spearman et al., 2019; Roger et al., 2021). HCV transmission primarily occurs through accidental needle sticks in medical settings, using injectable drugs, and receiving a blood transfusion before 1992, which was when blood screening became routine (Pietschmann and Brown, 2019; Hollande et al., 2020). Acute HCV cases account for about 15–20% of total cases, and post-acute HCV-infected patients have a 50–80% chance of developing a chronic infection (Narayanamurthy et al., 2021). If untreated, patients have a 20% risk of developing cirrhosis, of whom a small percentage may also develop hepatocellular carcinoma (Roger et al., 2021).

The WHO created a guide for the elimination of HCV by 2030, along with quantifiable targets (Warkad et al., 2018). The main factors required to eliminate HCV include increasing and strengthening outreach screening, improving prevention, and increasing access to treatment. Achieving HCV elimination requires scaling up rapid and accurate testing of populations worldwide, which inevitably places a heavy burden on developing countries (Foster et al., 2019; Dash et al., 2020). As a result, micro-elimination and cure have not been achieved because high-risk groups, including migrants from HCV-endemic countries, injecting drug users, prisoners, and men who have sex with men, and target groups, including hemophiliacs and those with a concurrent HIV infection (Hollande et al., 2020), require the development of rapid diagnostic testing methods and new therapeutic drugs. During the COVID-19 pandemic, less attention has focused on improving HCV testing. The timely detection of HCV, along with appropriate intervention measures, information dissemination, and outreach, is critical for an effective public health response to this virus (Javier et al., 2021).

HCV is a single-stranded, positive-stranded RNA virus belonging to the genus *Flaviviridae* (Houghton, 2009), which is composed of about 9,600 nucleotides. Due to its high degree of variation, vaccines and specific antiviral drugs are difficult to develop. Thus, an early diagnosis of HCV is critically important (Marcus et al., 2018). The gene encoding the capsid protein is comparatively conserved among different HCV genotypes, maintaining approximately 80% sequence identity (Adams et al., 2018). Current methods for detecting HCV include immunological and molecular biological tests. The immunological methods are generally utilized because of their simplicity and the convenience of automating batch operations. However, due to the long window of antigen and antibody detection, false negatives occur with some frequency (Kun et al., 2016; Llibre et al., 2019). Polymerase chain reaction (PCR)-based temperature-variable amplification technology is the most reliable HCV-RNA detection method and is considered the gold standard for HCV diagnosis (Fitzpatrick et al., 2018; Wang et al., 2021). While PCR-based tests are highly sensitive, they are expensive, are time-consuming, and require complex equipment, so they cannot be used for rapid point-of-care-testing (POCT) in low-resource areas with high HCV infection rates. Furthermore, the application of these tests may be restricted as a result of their detection potential.

In recent years, isothermal amplification methods have been studied and used for the detection of foodborne pathogens. Recombinase-aided amplification (RAA) is a novel isothermal amplification technology in which rapid amplification of DNA or RNA is achieved at constant temperatures. The entire RAA reaction is simple, rapid, accurate, power-saving, and convenient (Ivan and Ciara, 2017; Chen et al., 2018; Azmi et al., 2021). Other common isothermal amplification approaches include loop-mediated isothermal amplification (Notomi et al., 2000), rolling circle amplification (Blanco et al., 1989), recombinase polymerase amplification (Piepenburg et al., 2006), nucleic acid sequence-based amplification (Compton, 1991), and helicase-dependent amplification (Vincent et al., 2004). Isothermal amplification is commonly combined with a downstream portable result reading strategy to develop fast and convenient diagnostic testing methods, which are expected for use in remote areas and by developed countries that are performing mass screenings (Jia et al., 2018; Zhang et al., 2021). Of the isothermal amplification techniques, the RAA detection method is completed within 30 min at a constant temperature of 37 to 42°C. Thus, the technology is widely used for the rapid detection of viruses, bacteria, parasites, and other pathogens (Yan et al., 2014; Daher et al., 2016). RAA technology can also be combined with other novel detection methods, making pathogen detection more efficient and convenient. Its portable detection equipment also provides the possibility of POCT.

**Figure 1:**
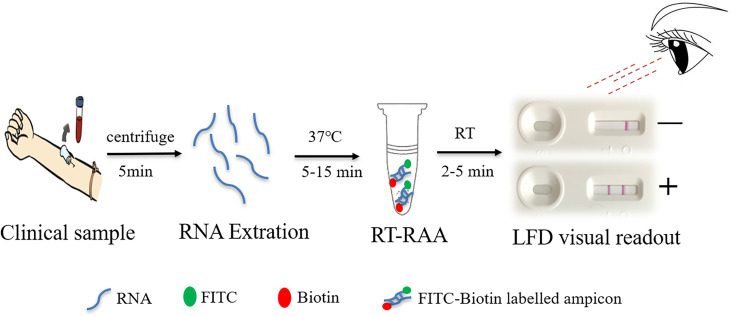
Schematic representation of the reverse transcription recombinase-aided amplification–lateral flow dipstick (RT-RAA-LFD) workflow for hepatitis C virus. RNA extraction for 15 min, RT-RAA reaction for 5 min, and LFD visual readout for 5 to 10 min. All work was performed within 30 min with minimal equipment requirements.

In this study, a rapid RT-RAA-LFD diagnostic platform was established for the detection of HCV, and its sensitivity, specificity, and stability were evaluated, providing a rapid, convenient, and accurate POCT strategy for HCV detection. A schematic figure representing the major steps of sample preparation for HCV detection using the RT-RAA-LFD method is shown in **Figure 1**. This method has great application potential because of its convenience, short detection time, and POCT application.

## Materials and Methods

### Clinical Samples

Two thousand clinical samples were collected at random from patients presenting to the outpatient clinic or physical examination center of the Henan Institute of Reproduction Health Science and Technology between January 2020 and January 2021. Each patient was given a health interview and asked to sign an informed consent. Of the 2,000 samples, 46 were found to be HCV-antibody-positive using automated chemiluminescence immunoassay. The blood pressure and serum indexes of the participant, the liver and kidney function as well as the results of B-ultrasound imaging examinations were normal. After fasting, 2 ml of blood was obtained from each patient and stored in a vacuum coagulation booster tube. Serum was then separated by centrifugation for 10 min at 3,500 *g*. The collected samples were deactivated by heating at 56°C for 30 min and immediately tested or stored at -20°C until use.

### Nucleic Acid Extraction

Nucleic acids were extracted from each clinical sample using TaKaRa MiniBEST Viral RNA/DNA Extraction Kit version 5.0 (Takara, Dalian, China) according to the manufacturer’s instructions. In brief, 200-μl serum samples were used for extraction. Total viral genomic RNA was eluted with 50 μl nuclease-free water and stored at -80°C until use. Based on the extracted HCV RNA genome, cDNAs were prepared with a PrimeScript™ RT reagent Kit (Takara, Dalian, China). The reaction conditions were 15 min at 37°C for reverse transcription and 5 s at 85°C for denaturation. The cDNAs were then stored at -80°C for later use.

### Primers and Plasmids

RAA primers targeting the HCV C/E1 genes were designed as described in the Twist-Dx (Maidenhead, UK) protocol. The RAA primers and the standard plasmid pUC57-pC/E1 (GenBank: AJ238799.1) used in this study were all synthesized by Sangon Biotech (Shanghai, China) (**Table 1**).

**Table 1:** Primer sequences used in this study.

Primer	Sequence (5′-3′)	Position (nt)
PCR-F	AAYYTDCCCGGTTGCTCTTTYTCTAT	842–867
PCR-R	TTCATCATCATRTCCCANGCCAT	1,309–1,331
RAA-F1	AGTACAGGACTGCAATTGCTCAATATATCC	1,241–1,270
RAA-R1	GCAAAGATAGCATCACAATCAGAACCTTAG	1,477–1,447
RAA-F2	CTTGGGATATGATGATGAACTGGTCACCTAC	1,297–1,327
RAA-R2	AAGAGTAGCATCACAATCAGAACCTTAGCC	1,474–1,445
LFA-F2	5′-FITC-CTTGGGATATGATGATGAACTGGTCACCTAC	
LFA-R2	5′-Biotin-AAGAGTAGCATCACAATCAGAACCTTAGCC	

### Basic PCR for HCV C/E1 Gene Detection

A 473-bp fragment in the C/E1 region of the HCV gene was amplified using conventional PCR methods and primers (PCR-F and PCR-R; **Table 1**). The region position of the three sets of primers targeted the C/E1 gene of the HCV 1b genome (GenBank: AJ238799.1). The 25-μl PCR reaction contained 1 μl of DNA template (10^8^ copies/μl to 1 copy/μl), 1 μl each of 10 μmol/L upstream and downstream primer, and 22 μl of Golden Star T6 Super PCR Mix (Beijing Tsingke Biotechnology Co., Ltd., China). The PCR program was conducted as follows: 95°C for 5 min, then 40 cycles at 95°C for 1 min, 47°C for 30 s, 72°C for 1 min, and a final extension at 72°C for 2 min. Amplicons were tested using 1% agarose gel electrophoresis.

### Quantitative Real-Time PCR

A commercial diagnostic kit for HCV RNA (PCR-fluorescence probing) (Daan Gene, Guangzhou, China) and an ABI 7500 fluorescence quantitative PCR system (Life Technologies, Foster City, CA, USA) were used for qPCR detection. The 25-μl qPCR reaction contained 20 μl of fluorescent PCR reaction mix and 5 μl of RNA template. qPCR was conducted as follows: 15 min at 37°C for reverse transcription and 5 s at 85°C for denaturation, followed by 45 cycles of 45 s at 93°C and 20 s at 55°C. The results were considered positive if the Ct value was <40, with an “S” type amplification curve, and considered negative if the Ct value was reported as undetermined, with fluorescent signal maintaining a background level.

### RT-RAA Assay

RT-RAA was performed using a basic RT-RAA nucleic acid amplification kit (nfo) (Zhongce, Jiangsu, China). The 50-μl RT-RAA reaction contained one RT-RAA lyophilized powder, 41.5 μl buffer A, 2 μl of 10 μM forward primer, and 2 μl of 10 μM reverse primer that was mixed and centrifuged. The mixture was added to the RT-RAA reaction tube containing lyophilized powder, and either 2 μl of RNA template or 2 μl of RNase-free water (negative control) was added next. The reaction was incubated in a 37°C water bath for 5 to 15 min. The amplified product was either purified with QIAquick PCR purification kit (Qiagen, USA) for agarose gel electrophoresis or diluted with PBST (1× phosphate-buffered saline with 0.1% Tween-20) for LFD readout.

### Preparation of Colloidal Gold and Dipstick

The dipstick was predominantly composed of the following parts: sample pad, backing card, conjugate pad, absorbent pad, and nitrocellulose membrane. The test strip test line (T line) and the quality control line (C line) were sprayed with BioDot XYZ3050 Dispense Platform (Irvin, CA, USA). The distance between these lines was about 5 mm. Streptavidin was fixed on the T line for specific binding with biotin groups. The antibody against rabbit/mouse antibody Fc fragment was fixed on the C line to intercept excess colloidal gold-labeled anti-FITC antibodies. If a target detection substance was present in the sample to be tested, it formed a colloidal gold marker–target detection substance–antibody complex, which gathered on the corresponding detection line, forming a colored precipitation line.

### RT-RAA Visual Readout With LFD

The RT-RAA-LFD primers (**Table 1**) included a 5′-FITC-labeled forward primer (LFD-F) and a 5′-biotin-labeled reverse primer (LFD-R). The 50-μl amplification system was configured according to the supplier’s instructions. The optimal reaction temperature and time for RT-RAA-LFD were achieved by individually performing a group of RT-RAA reactions at room temperature and at 35, 37, or 40°C in a water bath for 5, 10, or 15 min. After amplification, 20 μl RT-RAA reaction mix was then immediately diluted with 80 μl PBST for LFD readout. The result was read after the reaction was left to stand for 5 to 15 min. When the C line appeared in red, it indicated that the test results were valid. When the quality C line and T line were red at the same time, the result was interpreted as a positive result. When only the C line is red but the T line does not respond, it was interpreted as a negative result.

### Analytical Sensitivity and Specificity

RT-RAA-LFA sensitivity was evaluated using optimal reaction conditions. Tenfold serial dilutions of pUC57-pC/E1 were measured from 10^8^ copies to 1 copy/μl by RT-RAA-LFA and compared with PCR. RT-RAA-LFA specificity was estimated using serum samples from common infectious diseases, including hepatitis A virus (HAV), hepatitis B virus (HBV), HIV, syphilis, and human papillomavirus virus (HPV).

## Results

### Establishment of HCV RT-RAA-LFD and Optimization of Reaction Conditions

**Figure 2:**
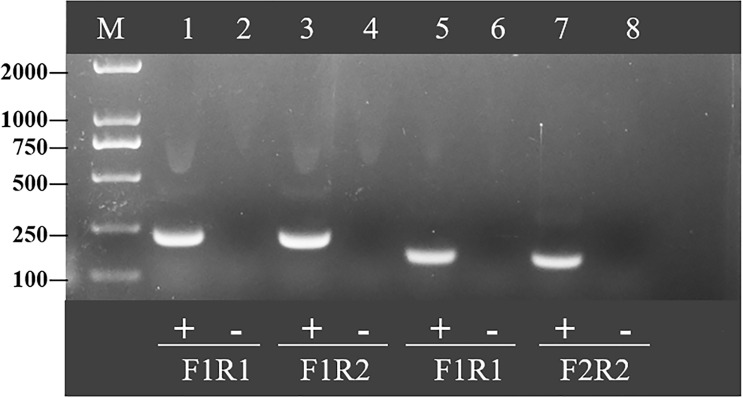
Optimal primer selection. Products amplified by different reverse transcription recombinase-aided amplification primers were analyzed by 1.5% agarose gel electrophoresis to select the optimal primer pairs. M is a 2,000-bp DNA marker. The primers and templates used in lanes 1–8 are as follows: 1, HCV-RAA-F1/R1 + HCV; 2, HCV-RAA-F1/R1 + ddH_2_O; 3, HCV-RAA-F1/R2 + HCV; 4, HCV-RAA-F1/R2 + ddH_2_O; 5, HCV-RAA-F2/R1 + HCV; 6, HCV-RAA-F2/R1 + ddH_2_O; 7, HCV-RAA-F2/R2 + HCV; 8, HCV-RAA-F2/R2 + ddH_2_O.

To determine the best primer combination, RAA amplification was conducted as described above. The four pairs of primer combinations produced brighter bands and a single band by agarose gel electrophoresis (**Figure 2**). Since a smaller amplification product increases the efficiency of amplification, the primer pair with the smallest product, RAA-F2 and RAA-R2, was selected for subsequent experiments.

**Figure 3:**
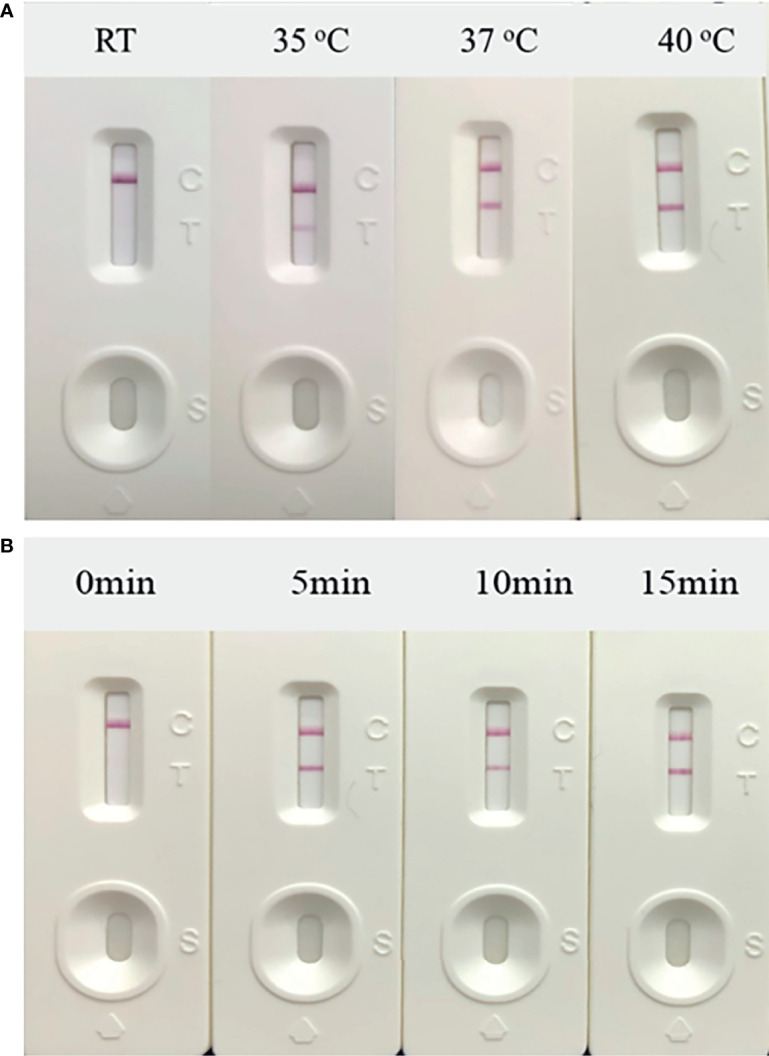
Optimization of reaction conditions. Using the recombinant plasmid PUC57-pC/E1 as a template to optimize the temperature conditions of hepatitis C virus (HCV) reverse transcription recombinase-aided amplification (RAA) strip. C is the control line, T is the test line, and N is negative control. **(A)** Determination of the optimal reaction temperature range of the HCV RAA strip. **(B)** Detection of the optimized amplification time.

Next, the temperature of the RT-RAA amplification system was optimized. The amplification and incubation were developed at 35 to 40°C for 10 min, and the results indicated that, when the amplification temperature was 37°C, the detection line color of the positive control was observed (**Figures 3A**). At 35°C, the color of the T line was weak, which was not significant, and a temperature of 40°C was considered too high for on-site experiments. Thus, 37°C was selected as the amplification temperature.

The amplification incubation time was also optimized. The amplification system was incubated in a 37°C water bath for 5, 10, and 15 min. After 5 min of incubation, the color of the T line started to turn red (**Figure 3B**). Thus, the final RT-RAA-LFD conditions used in this study included amplification at 37°C for 5 min and incubation at room temperature for 10 to 15 min to ensure that the results could be read.

### Sensitivity and Specificity of the RT-RAA-LFD Assay

**Figure 4:**
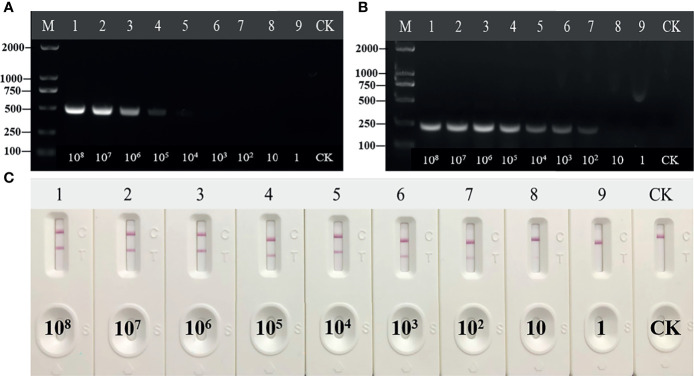
Sensitivity of reverse transcription recombinase-aided amplification (RT-RAA), RT-RAA-lateral flow dipstick, and PCR for plasmid pUC57-pC/E1. **(A)** Agarose gel electrophoresis of PCR. M, DL2000DNA marker; 1 to 9, decimal dilutions of plasmid pUC57-pC/E1 from 10^8^ copies/ml to 1 copy/ml. **(B)** Agarose gel electrophoresis of basic RAA. **(C)** RAA-LFA visual readout. CK, negative control with double-distilled water.

The HCV standard plasmid was diluted into different concentration gradients (10^8^ copies/μl–1 copy/μl) for RT-RAA-LFD detection. While the detection limit of conventional PCR was 10^4^ copies/μl (**Figure 4C**), the detection limit of RAA was 10^2^ copies/μl (**Figure 4A**). The sensitivity and detection limit of RT-RAA-LFD were 100 and 10 times higher (reaching 10 copies/μl), respectively, than those of basic RAA (**Figure 4B**). Each assay was repeated three times.

**Figure 5:**
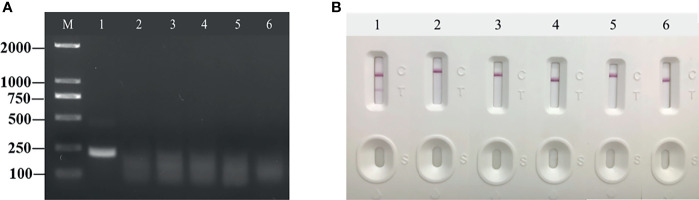
Specificity of reverse transcription recombinase-aided amplification (RT-RAA) and RT-RAA-lateral flow dipstick for common porcine diseases. **(A)** Agarose gel electrophoresis of RT-RAA. M, DL 2000 DNA marker; 1, HCV; 2, HAV; 3, HBV; 4, HIV; 5, syphilis; and 6, HPV. **(B)** RT-RAA-LFA visual readout. 1, HCV; 2, HAV; 3, HBV; 4, HIV; 5, syphilis; and 6, HPV.

In the specificity tests, the other four of the five inspections for infectious diseases, HAV, HBV, HIV, and syphilis, and the HPV standard plasmid were used as specific test samples. These samples only developed color at the quality control line and were defined as negative. RT-RAA-LFD showed adequate specificity and no cross-reactivity with other viruses (**Figures 5A, B**).

### Stability Assay of RT-RAA-LFD

**Figure 6:**
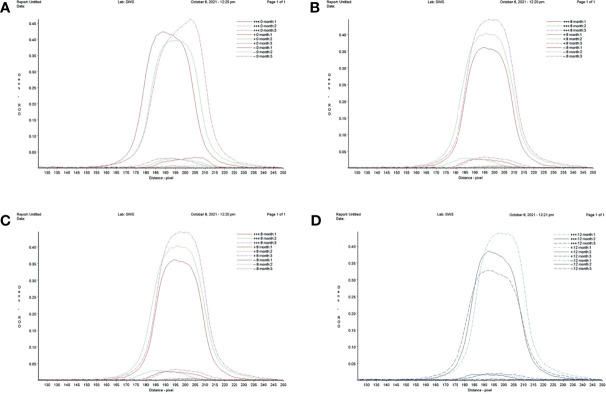
Repeatability and stability of reverse transcription recombinase-aided amplification–lateral flow dipstick. **(A)** Screening of lateral flow dipstick’s test lines at 0 month. **(B)** Screening of lateral flow dipstick’s test lines at 4 months. **(C)** Screening of lateral flow dipstick’s test lines at 8 months. **(D)** Screening of lateral flow dipstick’s test lines at 12 months. +++, strong positive sample, 10^5^ copies/ml; +, weak positive sample 10 copies/ml; -, negative sample, double-distilled water.

The RT-RAA-LFD stability assay was performed at 0, 4, 8, and 12 months after the sensitivity assay by testing in triplicate strong positive samples (10^5^ copies/μl; +++) and weak positive samples (10 copies/μl; +) of standard plasmid pUC57-pC/E1 and negative samples (double-distilled water; -) at three times each (**Figure 6**). LFD was stored at room temperature and in dry conditions. The relative optical density value of the LFD T line was recorded using the TSR3000 filmstrip reader (BioDot). As shown in **Table 2**, the coefficient of variance (Cv) values for strong positive, weak positive, and negative samples at the four time points were 4.43, 3.51, and 9.67%, respectively, which were all lower than 10%. This indicated that the RT-RAA-LFD method had adequate detection stability within 1 year.

**Table 2:** Readability and stability assay of RT-RAA-LFA.

Time point (month) sample	0	4	8	12	Cv value
	Average relative optical density of three repeat tests + SD				
10^5^ copies/μl, +++	960.3892 ± 169.97	871.9121 ± 71.21	897.6027 ± 240.09	880.2978 ± 188.98	4.43%
10 copies/μl, +	61.8697 ± 5.81	58.3425 ± 19.23	57.3541 ± 13.68	57.7576 ± 7.69	3.51%
Double-distilled water, -	7.4354 ± 2.4073	6.2999 ± 1.0155	7.8315 ± 0.51	7.7612 ± 0.95	9.67%

### Clinical Sample Analysis Using Quantitative Real-Time PCR

Following positive serology, the gold standard confirmation test of HCV infection was the detection of HCV RNA by qPCR. HCV-RNA >1.0 × 10^3^ IU/ml was considered positive, HCV-RNA quantification <1.0 × 10^6^ IU/ml was considered a low viral load, and HCV-RNA quantification ≥1.0 × 10^6^ IU/ml was considered a high viral load. The RNA extracted from 46 clinical samples were tested using the commercially available qPCR kit. The qPCR results showed that nine of the 46 clinical samples were positive, with a viral load ranging from 2.81 × 10^8^ IU/ml to 8.4 × 10 IU/ml. HCV RNA-positive patients accounted for 19.56% (9/46), while HCV RNA-negative patients accounted for 80.44% (37/46) (**Table 3**).

**Table 3:** Analysis of quantitative detection results of HCV-RNA in 46 patients.

Quantification of HCV-RNA (IU/ml)	Number of samples	Proportion (%)
(-) <1.0 × 10^3^	37	80.44
(+) 10^3^–<1.0 × 10^6^	6	13.04
(+) ≥1.0 × 10^6^	3	6.52
Total	46	100%

### Performance of the RT-RAA-LFD Assay on Clinical Samples

**Figure 7:**
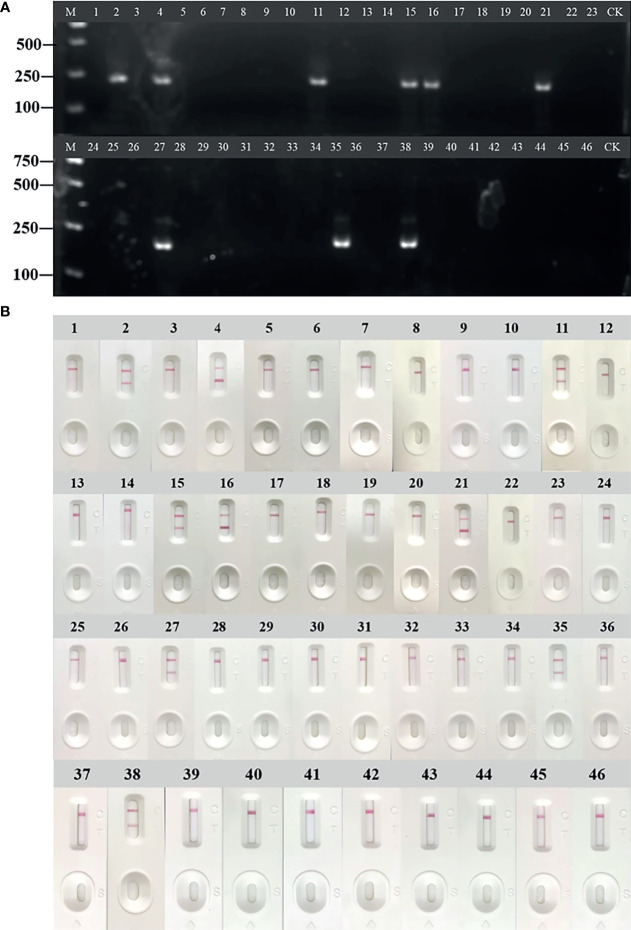
Evaluation of reverse transcription recombinase-aided amplification (RT-RAA) and RT-RAA-lateral flow dipstick (LFD) in RNA extracted from clinical samples. **(A)** Agarose gel electrophoresis of RT-RAA. **(B)** RT-RAA-LFD visual readout. M, DL2000 DNA marker; 1 to 46, blood sample number; CK, negative control with double-distilled water.

Forty-six cases of anti-HCV antibody-positive clinical samples were used to evaluate the performance of RT-RAA-LFD. The clinical samples were detected by RT-RAA, RT-RAA-LFD. The results of RT-RAA, RT-RAA-LFD on nine clinical serum samples were consistent with the results of the traditional qPCR, indicating that this method has the potential for clinical application (**Figures 7A, B** and **Table 4**). In addition, the positive samples containing a high viral load showed color very quickly (2 to 3 min) at the T lines.

**Table 4:** Detection in clinical samples by RT-RAA, RT-RAA-LFA and qPCR.

Assay	Number of samples	
	Positive	Negative
RT-RAA	9	37
RT-RAA-LFA	9	37
qPCR	9	37

## Discussion

Infectious diseases caused by viruses, bacteria, fungi, and other pathogens are an inevitable challenge to global health and food security (Chevaliez, 2019). During the COVID-19 pandemic, most secondary and above-detection institutions were equipped with real-time quantitative PCR machines. However, PCR detection technology is expensive and has strict requirements for the detection, collection, and storage of samples so that it is not suitable for widespread use. The disruption to the existing medical service system by COVID-19 led to a reduction in routine HCV antibody screening and a delay in clinical care and treatment (Wan et al., 2020; Yoo et al., 2021). The later detection of HCV infections that have occurred during the COVID-19 pandemic has resulted in higher morbidity and mortality. To prevent the spread of disease and protect human populations, rapid POCT for human diseases has played an increasingly important role. Rapid pathogenic diagnosis methods are critical to animal disease control and prevention, public health safety, and other issues (Patchsung et al., 2020; Zhao et al., 2021).

In this study, a novel and complete POCT method for HCV detection, combined with RT-RAA and LFD, was described to have demonstrated good sensitivity and specificity. The detection limit for detecting synthesized plasmids reached 10 copies/μl, and no cross-reactivity was seen with HAV, HBV, HIV, syphilis, and HPV, showing adequate specificity. With RNA extracted from clinical samples, the RT-RAA-LFD method showed 100% concordance with qPCR. However, RT-RAA-LFD could be completed with 5-min amplification and 5–15-min LFD readout times (clinical samples with high RNA concentrations only required 2 to 3 min), indicating that this method was simpler and more efficient than qPCR. Thus, RT-RAA-LFD is a new method of HCV diagnosis that shows good results using exploratory tests. One limitation of this study is the small number of HCV-positive samples used for the evaluation. A field evaluation of this assay with a larger sample size is necessary. Clinical trial protocols will need to be developed and institutions selected for large-scale testing. In addition, the feasibility and accuracy of the RT-RAA-LFD method will need to be evaluated in remote areas.

The RT-RAA experimental method established in this study is based on the use of commercial nucleic acid extraction kits to extract viral genomic RNA, which requires significant preparation costs and expensive instruments like centrifuges and automated RNA extractors. While the traditional qPCR method costs about $5 dollars per test, the cost of an RT-RAA-LFD reaction is about $3 dollars, A significant limitation of this method is that the enzymes must be purchased in a commercial kit. Future studies will focus on independent production from recombinant enzymes, which should reduce the cost and make the test more accessible for POCT. One of the main obstacles for COVID-19 testing is also the extraction of viral RNA, which slows the detection (Min et al., 2021). In recent years, a number of simple RNA extraction-free methods have been used for clinical testing, which play an important role in disease detection and promote the development of rapid-detection reagents (Azmi et al., 2021). However, these methods have their own limitations and shortcomings. In future research, diagnostics may be simplified by preparing microfluidic samples on a chip and implementing an aqueous two-phase-system-based sample preparation and/or paper-based filtration. Efforts must be made to reduce the limitations of the current isothermal amplification methods, like non-specific binding and false positives. With the rapid development of this technology, traditional RAA technology may be combined with other novel technologies, such as real-time fluorescent RAA, quantum dots, CRISPR techniques, electrochemical sensors, and other auxiliary methods, to determine results more conveniently and intuitively. If quantitative detection is required, fluorescent strips may be used for the semi-quantitative rapid detection of diseases. At the same time, the application prospect of this technology for the detection of pathogenic microorganisms has greatly expanded.

In conclusion, this study has shown that RT-RAA-LFD is a highly sensitive and specific method for HCV detection. This method is fast, convenient, and instrument-free, so it may also be performed in remote areas.

## Data Availability Statement

The original contributions presented in the study are included in the article/supplementary material. Further inquiries can be directed to the corresponding authors.

## Ethics Statement

Ethics committee approval was obtained from the Institutional Ethics Committee of Henan Institute of Reproduction Health Science prior to the commencement of the study (approval number EC-258 20200713-1012).

## Author Contributions

All the authors involved played an important role and contributed to the article. All authors approved the submitted version. HW, YuZ, GZ, and AW contributed to the research design. CL and XZ analyzed the data. HW, ML, JZ, and YC provided clinical samples and conducted the molecular biology experiments. YuZ, HL, CL, XZ, YiZ, and CX performed the experiments and provided study supervision. HW and YuZ wrote the manuscript.

## Funding

This work was supported by grants from the Science and Technology Project of Henan Province (no. 212102310180), the Open Project of National Health Commission Key Laboratory of Birth Defects Prevention (no. ZD202106), and the Scientific and Technological Innovation Team Plan of Henan Province (19IRTSTHN006).

## Conflict of Interest

The authors declare that the research was conducted in the absence of any commercial or financial relationships that could be construed as a potential conflict of interest.

## Publisher’s Note

All claims expressed in this article are solely those of the authors and do not necessarily represent those of their affiliated organizations, or those of the publisher, the editors and the reviewers. Any product that may be evaluated in this article, or claim that may be made by its manufacturer, is not guaranteed or endorsed by the publisher.

## References

[B1] Adams L. M. Balderson B. Packett B. N. (2018a). Meeting the Challenge: Hepatitis C Virus and HIV Care Experiences Among HIV Specialty Providers. AIDS Patient Care STDS 32 (8), 314–320. doi: 10.1089/apc.2018.0006 30067406

[B2] Azmi I. Faizan M. I. Kumar R. Raj Yadav S. Chaudhary N. Kumar Singh D.. (2021). A Saliva-Based RNA Extraction-Free Workflow Integrated With Cas13a for SARS-CoV-2 Detection. Front. Cell. Infection Microbiol. 11. doi: 10.3389/fcimb.2021.632646 PMC8009180 33796478

[B3] Blanco L. Bernad A. Lázaro J. M. Martín G. Garmendia C. Salas M. (1989). Highly Efficient DNA Synthesis by the Phage Φ 29 DNA Polymerase: Symmetrical Mode of DNA Replication. J. Biol. Chem. 264 (15), 8935–8940. doi: 10.1016/S0021-9258(18)81883-X 2498321

[B4] Chen J. S. Ma E. Harrington L. B. Da C. M. Tian X. Palefsky J. M.. (2018). CRISPR-Cas12a Target Binding Unleashes Indiscriminate Single-Stranded DNase Activity. Science 360 (6387), 436–439. doi: 10.1126/science.aar6245 29449511 PMC6628903

[B5] Chevaliez S. (2019). Strategies for the Improvement of HCV Testing and Diagnosis. Expert Rev. Anti Infect. Ther. 17 (5), 341–347. doi: 10.1080/14787210.2019.1604221 30950298

[B6] Compton J. (1991). Nucleic Acid Sequence-Based Amplification. Nature 350 (6313), 91–92. doi: 10.1038/350091a0 1706072

[B7] Daher R. K. Stewart G. Boissinot M. Bergeron M. G. (2016). Recombinase Polymerase Amplification for Diagnostic Applications. Clin. Chem. 62 (7), 947–958. doi: 10.1373/clinchem.2015.245829 27160000 PMC7108464

[B8] Dash S. Aydin Y. Widmer K. E. Nayak L. (2020). Hepatocellular Carcinoma Mechanisms Associated With Chronic HCV Infection and the Impact of Direct-Acting Antiviral Treatment. J. Hepatocell Carcinoma 7, 45–76. doi: 10.2147/JHC.S221187 32346535 PMC7167284

[B9] Fitzpatrick T. Pan S. W. Tang W. Guo W. Tucker J. D. (2018). HBV and HCV Test Uptake and Correlates Among Men Who Have Sex With Men in China: A Nationwide Cross-Sectional Online Survey. Sex Transm Infect. 94 (7), 502–507. doi: 10.1136/sextrans-2018-053549 29779005 PMC6195464

[B10] Foster G. R. Dore G. J. Wang S. Grebely J. Sherman K. E. Baumgarten A.. (2019). Glecaprevir/pibrentasvir in Patients With Chronic HCV and Recent Drug Use: An Integrated Analysis of 7 Phase III Studies. Drug Alcohol Depend 194, 487–494. doi: 10.1016/j.drugalcdep.2018.11.007 30529905

[B11] Hollande C. Parlati L. Pol S. (2020). Micro-Elimination of Hepatitis C Virus. Liver Int. 40 Suppl 1, 67–71. doi: 10.1111/liv.14363 32077601

[B12] Houghton M. (2009). Discovery of the Hepatitis C Virus. Liver Int. 29 (Suppl 1), 82–88. doi: 10.1111/j.1478-3231.2008.01925.x 19207970

[B13] Ivan M. L. Ciara K. O. (2017). Recombinase Polymerase Amplification: Basics, Applications and Recent Advances. Trends Analyt Chem. 98, 19–35. doi: 10.1016/j.trac.2017.10.015 PMC7112910 32287544

[B14] Javier C. Álvaro D. Joaquín C. (2021). HCV Detection is Possible During SARS CoV-2 Testing; and Throughout COVID-19 Vaccination? J. Hepatol. 75 (2), 486–487. doi: 10.1016/j.jhep.2021.04.043 33971225 PMC8103740

[B15] Jia L. Joanne M. Felix V. S. (2018). Review: A Comprehensive Summary of a Decade Development of the Recombinase Polymerase Amplification. Analyst. 144 (1), 31–67. doi: 10.1039/c8an01621f 30426974

[B16] Kun W. Daoqing F. Yaqing L. Shaojun D. (2016). Cascaded Multiple Amplification Strategy for Ultrasensitive Detection of HIV/HCV Virus DNA. Biosens. Bioelectron. 87, 116–121. doi: 10.1016/j.bios.2016.08.017 27526400

[B17] Llibre A. Shimakawa Y. Duffy D. (2019). Potential Utility of the Genedrive Point-of-Care Test for HCV RNA Detection. Gut. 68 (10), 1903–1904. doi: 10.1136/gutjnl-2018-317218 30244200 PMC6839793

[B18] Marcus J. L. Hurley L. B. Chamberland S. Champsi J. H. Gittleman L. C. Korn D. G.. (2018). No Difference in Effectiveness of 8 vs 12 Weeks of Ledipasvir and Sofosbuvir for Treatment of Hepatitis C in Black Patients. Clin. Gastroenterol. Hepatol. 16 (6), 927–935. doi: 10.1016/j.cgh.2018.03.003 29535057 PMC5962408

[B19] Massimo C. Vincenzo B. (2018). HCV Therapy and Risk of Liver Cancer Recurrence: Who to Treat? Nat. Rev. Gastroenterol. Hepatol. 15 (7), 392–393. doi: 10.1038/s41575-018-0018-5 29752455

[B20] Min L. Fangfei Y. Lu S. Xiuhai M. Fan L. Chunhai F.. (2021). Nucleic Acid Tests for Clinical Translation. Chem. Rev. 121 (17), 10469–10558. doi: 10.1021/acs.chemrev.1c00241 34254782

[B21] Narayanamurthy V. Jeroish Z. E. Bhuvaneshwari K. S. Samsuri F. (2021). Hepatitis C Virus (HCV) Diagnosis via Microfluidics. Analytical Methods 13 (6), 74–763. doi: 10.1039/d0ay02045a 33511975

[B22] Notomi T. Okayama H. Masubuchi H. Yonekawa T. Watanabe K. Amino N.. (2000). Loop-Mediated Isothermal Amplification of DNA. Nucleic Acids Res. 28 (12), E63. doi: 10.1093/nar/28.12.e63 10871386 PMC102748

[B23] Patchsung M. Jantarug K. Pattama A. Aphicho K. Suraritdechachai S. Meesawat P.. (2020). Clinical Validation of a Cas13-Based Assay for the Detection of SARS-CoV-2 RNA. Nat. Biomed. Eng. 4 (12), 1140–1149. doi: 10.1038/s41551-020-00603-x 32848209

[B24] Piepenburg O. Williams C. H. Stemple D. L. Armes N. A. (2006). DNA Detection Using Recombination Proteins. PloS Biol. 4 (7), e204. doi: 10.1371/journal.pbio.0040204 16756388 PMC1475771

[B25] Pietschmann T. Brown R. J. P. (2019). Hepatitis C Virus. Trends Microbiol. 27 (4), 379–380. doi: 10.1016/j.tim.2019.01.001 30709707

[B26] Roger S. Ducancelle A. Le Guillou-Guillemette H. Gaudy C. Lunel F. (2021). HCV Virology and Diagnosis. Clinics Res. Hepatol. Gastroenterol. 45 (3), 101626. doi: 10.1016/j.clinre.2021.101626 33636428

[B27] Spearman C. W. Dusheiko G. M. Hellard M. Sonderup M. (2019). Hepatitis C. Lancet 394 (10207), 1451–1466. doi: 10.1016/S0140-6736(19)32320-7 31631857

[B28] Vincent M. Xu Y. Kong H. (2004). Helicase-Dependent Isothermal DNA Amplification. EMBO Rep. 5 (8), 795–800. doi: 10.1038/sj.embor.7400200 15247927 PMC1249482

[B29] Wang Y. Jie W. Ling J. Yuanshuai H. (2021). HCV Core Antigen Plays an Important Role in the Fight Against HCV as an Alternative to HCV-RNA Detection. J. Clin. Lab. Anal. 35 (6), e23755. doi: 10.1002/jcla.23755 33788295 PMC8183919

[B30] Wan D. Y. Luo X. Y. Dong W. Zhang Z. W. (2020). Current Practice and Potential Strategy in Diagnosing COVID-19. Eur. Rev. Med. Pharmacol. Sci. 24 (8), 4548–4553. doi: 10.26355/eurrev_202004_21039 32374007

[B31] Warkad S. D. Nimse S. B. Song K. S. Kim T. (2018). HCV Detection, Discrimination, and Genotyping Technologies. Sensors (Basel) 18 (10), 3423. doi: 10.3390/s18103423 PMC6210034 30322029

[B32] World Health Organization. (2017). Guidelines on Hepatitis B and C Testing. Geneva: World Health Organization.

[B33] Yan L. Zhou J. Zheng Y. Gamson A. S. Roembke B. T. Nakayama S.. (2014). Isothermal Amplified Detection of DNA and RNA. Mol. Biosyst. 10 (5), 970–1003. doi: 10.1039/c3mb70304e 24643211

[B34] Yoo H. M. Kim I. Kim S. (2021). Nucleic Acid Testing of SARS-CoV-2. Int. J. Mol. Sci. 22 (11), 6150. doi: 10.3390/ijms22116150 34200331 PMC8201071

[B35] Zhang Y. Li Q. Guo J. Li D. Wang L. Wang X.. (2021). An Isothermal Molecular Point of Care Testing for African Swine Fever Virus Using Recombinase-Aided Amplification and Lateral Flow Assay Without the Need to Extract Nucleic Acids in Blood. Front. Cell. Infection Microbiol. 11. doi: 10.3389/fcimb.2021.633763 PMC8010139 33816338

[B36] Zhao L. Wang J. Sun X. X. Wang J. Chen Z. Xu X.. (2021). Development and Evaluation of the Rapid and Sensitive RPA Assays for Specific Detection of Salmonella Spp. In Food Samples. Front. Cell. Infection Microbiol. 11. doi: 10.3389/fcimb.2021.631921 PMC7946851 33718280

